# Comparative analysis of new mScarlet-based red fluorescent tags in *Caenorhabditis elegans*

**DOI:** 10.1093/genetics/iyae126

**Published:** 2024-08-06

**Authors:** Wen Xi Cao, Daniel M Merritt, Karinna Pe, Michael Cesar, Oliver Hobert

**Affiliations:** Department of Biological Sciences, Columbia University, Howard Hughes Medical Institute, New York, NY 10027, USA; Department of Biological Sciences, Columbia University, Howard Hughes Medical Institute, New York, NY 10027, USA; Department of Biological Sciences, Columbia University, Howard Hughes Medical Institute, New York, NY 10027, USA; Department of Biological Sciences, Columbia University, Howard Hughes Medical Institute, New York, NY 10027, USA; Department of Biological Sciences, Columbia University, Howard Hughes Medical Institute, New York, NY 10027, USA

**Keywords:** *Caenorhabditis elegans*, fluorophore, marker, RFP

## Abstract

One problem that has hampered the use of red fluorescent proteins in the fast-developing nematode *Caenorhabditis elegans* has been the substantial time delay in maturation of several generations of red fluorophores. The recently described mScarlet-I3 protein has properties that may overcome this limitation. We compare here the brightness and onset of expression of CRISPR/Cas9 genome-engineered mScarlet, mScarlet3, mScarlet-I3, and GFP reporter knock-ins. Comparing the onset and brightness of expression of reporter alleles of *C. elegans golg-4*, encoding a broadly expressed Golgi resident protein, we found that the onset of detection of mScarlet-I3 in the embryo is several hours earlier than older versions of mScarlet and comparable to GFP. These findings were further supported by comparing mScarlet-I3 and GFP reporter alleles for *pks-1*, a gene expressed in the CAN neuron and cells of the alimentary system, as well as reporter alleles for the pan-neuronal, nuclear marker *unc-75*. Hence, the relative properties of mScarlet-I3 and GFP do not depend on cellular or subcellular context. In all cases, mScarlet-I3 reporters also show improved signal-to-noise ratio compared to GFP.

## Teaser

Fluorescent proteins are instrumental in the visualization of gene expression, cell and tissue morphology and developmental processes in the transparent roundworm Caenorhabditis elegans. However, existing red fluorescent proteins (RFP), have very slow maturation times, resulting in significant time before an expressed gene or reporter can be visualized. In this paper, the authors test newly developed RFPs mScarlet3 and mScarlet-I3 in the worm using three different CRISPR-based reporters. They find that these new RFPs offer increased brightness and greatly improved maturation speed, demonstrating the usefulness of these tools to the *C. elegans* community.

## Introduction

mScarlet is a commonly used fluorophore that provides many advantages over previous red fluorescent proteins (RFPs) (Bindels *et al.* 2017). In *Caenorhabditis elegans*, worm codon-optimized mScarlet (also known as wrmScarlet) has been shown to be 8-fold brighter than TagRFP-T, to show improved signal-to-noise ratio, and to be more red-shifted than TagRFP-T (El Mouridi *et al.* 2017). These properties maximize signal while limiting bleed-through when used in conjunction with a green or yellow-green fluorophore such as GFP or mNeonGreen (El Mouridi *et al.* 2017). Furthermore, mScarlet is specifically designed to be monomeric, and does not pass through a green immature intermediate like other red fluorophores, such as tdTomato or mCherry (Wiehler *et al.* 2001; Verkhusha *et al.* 2004; Wachter *et al.* 2010; Bindels *et al.* 2017).

However, a lingering issue that plagues most RFPs used in *C. elegans*, including mScarlet, is their slow maturation time. Green fluorophores, including GFP, mEGFP, and mNeonGreen, are both rapidly maturing and bright, with characterized maturation times of around 30 min or less at 37°C in cell culture [(Balleza *et al.* 2018); fpbase.org]. In contrast, many RFPs display maturation times on the order of several hours at 37°C in cell culture [(Bindels *et al.* 2017; Balleza *et al.* 2018); fpbase.org]. Exceptions to this are mCherry, which is not ideal due its propensity to aggregate and induce toxicity (Katayama *et al.* 2008; Melentijevic *et al.* 2017); and the mScarlet-derived mScarlet-I, which is reported to have a much faster maturation at the expense of decreased brightness (Bindels *et al.* 2017). In the rapidly developing model organism *C. elegans*, where key developmental events occur over time scales of very few hours (e.g. the cell cycle time in the developing embryo is on the order of ∼20 min, and the complete embryo hatches after ∼800 min of development), delayed fluorophore maturation impedes efforts to visualize rapid changes in gene expression. Also, the fluorophore-based visualization of cellular morphology changes often necessitates the availability of rapidly maturing fluorophores. For example, given that many cell type-selective drivers only become active during terminal differentiation, slowly maturing RFPs cannot be used to visualize processes, such as neurite outgrowth, which only happen at or around the time of terminal differentiation.

Recently, additional rapidly maturing derivatives of mScarlet have been described (Gadella *et al.* 2023). These RFPs, namely mScarlet3 and mScarlet-I3, have been characterized in cells lines to have much improved maturation properties with minimal impact on brightness, and to retain nearly identical excitation/emission spectra as mScarlet. When characterized in vivo and in mammalian cell culture, mScarlet3 was found to be brighter while maturing 4 times faster than mScarlet (Gadella *et al.* 2023). mScarlet-I3 is the fastest maturing RFP characterized with only a slight decrease in relative brightness when compared to mScarlet (Gadella *et al.* 2023). Here, we codon-optimize these new RFPs, generate a series of reporter alleles via CRISPR/Cas9-mediated engineering into the *C. elegans* genome, and compare their intensity and dynamics in vivo against commonly used mScarlet and GFP reporters.

## Materials and methods

### *C. elegans* strains and maintenance

*C. elegans* strains were cultivated at 20°C on plates containing Nematode Growth Media seeded with *E. coli* strain OP50.

Previously published strains used in this study:

OH19204 *golg-4(ot1508[gfp::golg-4])* (Leyva-Diaz *et al*. 2024)

PHX6499 *unc-75(syb6499[gfp::unc-75]*) (Leyva-Diaz *et al*. 2024)

Strains generated for this study:

PHX6547 *golg-4(syb6547[wrmScarlet::golg-4)*

OH19205 *golg-4(ot1509[mScarlet3::golg-4])*

OH19206 *golg-4(ot1510[mScarlet-I3::golg-4])*

OH19124 *pks-1(ot1488[pks-1::SL2::mScarlet-I3::h2b])*

OH19125 *pks-1(ot1489[pks-1::SL2::gfp::h2b])*

*OH19299 *unc-75(ot1539[mScarlet-I3::unc-75])**

### Fluorophore design and transgenics

Sequences of the fluorophore tags used can be found in Supplementary File 1. *C. elegans* codon-optimized GFP coding sequence containing 3 introns was amplified from pPD95.75 plasmid. Protein sequences of mScarlet3 and mScarlet-I3 were obtained from fpbase.org and (Gadella *et al.* 2023), and reverse translated. The coding sequence were codon-optimized for *C. elegans* expression with 2 introns added, using sequences recommended by the MPI *C. elegans* codon adapter (Redemann *et al.* 2011), and synthesized by IDT. Fluorophores were knocked into the respective genomic loci using CRISPR/Cas9-based genome editing protocol described in Eroglu *et al.* (2023), using the *dpy-10* co-conversion marker and reagents from IDT. PHX6547 and PHX6499 were generated by SunyBiotech. The insert and flanking sequences were verified by Sanger sequencing.

### Embryo collection and staging

Embryos for microscopy imaging were obtained either by directly picking off the plates onto microscope slides with agar pads (for later stages), dissection of gravid adults (for earlier stages), or collected in large numbers with a gentle egg prep. To dissect, gravid adults were transferred into a droplet of M9 on a glass slide, and cut through the midbody using an 18-gauge needle. Released embryos were aspirated into a microcapillary pipette and transferred directly onto agar pad slides by mouth pipette. For the gentle egg prep, worms were washed off nearly starved plates enriched for embryos and gravid adults with M9 buffer and pelleted by centrifugation at 2,000×g for 1 min. Worms were treated in a mixture of 500 μl distilled water, 500 μl household bleach, and 50 μl of 10 M sodium hydroxide for 4–5 min with shaking, or just until adult bodies break apart and eggs are released. Embryos are pelleted by centrifugation and washed with 1 ml of M9 3 times, then resuspended in M9 buffer and spotted onto agar pad slides for imaging. Individual embryo stages were visualized using different interference contrast (DIC) microscopy at the time of fluorescent imaging.

### Microscopy

Adult worms were mounted on 5% agar pads and anesthetized with 50 mM sodium azide in M9 buffer. Embryos were obtained as described above, and mounted in M9 buffer without sodium azide. Images were acquired on a Zeiss LSM980 confocal microscope using the ZEN Blue image acquisition software with preset excitation and emission parameters for EGFP and mScarlet (*golg-4* and *unc-75* strains). Specifically, mScarlet, mScarlet3, and mScarlet-I3 strains were imaged using identical parameters of 561 nm excitation laser diode at 10%, and 561–694 nm detection wavelength on a GaAsP-PMT detector with 650 V gain and 2× averaging. GFP was imaged using 488 nm excitation laser diode at 5% (for *golg-4*) or 10% (*for unc-74*), and 490–579 nm detection wavelength on a GaAsP-PMT detector with 700 V gain and 2× averaging. *pks-1* strains were imaged using the Zeiss Axioimager Z2 light microscope with the Colibri 7 LED light source and captured using a Hamamatsu ORCA-Fusion digital camera (C14440), also with ZEN Blue. Specifically, mScarlet-I3 was imaged using 567 nm LED module at 100% intensity with 540–570 nm excitation filters, 579–604 nm emission filters, and 200 ms exposure time. GFP was imaged using 475 nm LED module at 30% intensity with 450–490 nm excitation filters, 500–550 emission filters, and 50 ms exposure time. For each image, Z-stacks of 1 μm thickness were obtained for the entire sample, and the medial section was later chosen for analysis.

### Image quantification and analyses

For embryos, representative images of the approximate medial section of embryos acquired at each stage are shown. For adult expression, fluorescence intensity of a single *Z* plane imaged through the middle of the adult grinder, visualized by DIC, was chosen for quantification. For representative images of UNC-75 expression in adults, maximum intensity projections were shown to visualize all neuron nuclei. Images were analyzed using ImageJ FIJI (Schindelin *et al.* 2012), and quantifications were visualized using GraphPad PRISM 8.3.0. For each fluorophore, average fluorescence intensity of the region of interest of at least 10 individual animals were quantified, plotted, and the median values were compared with a two-tailed t test.

## Results

### mScarlet3 is an exceptionally bright fluorophore in *C. elegans*

**Fig. 1.**
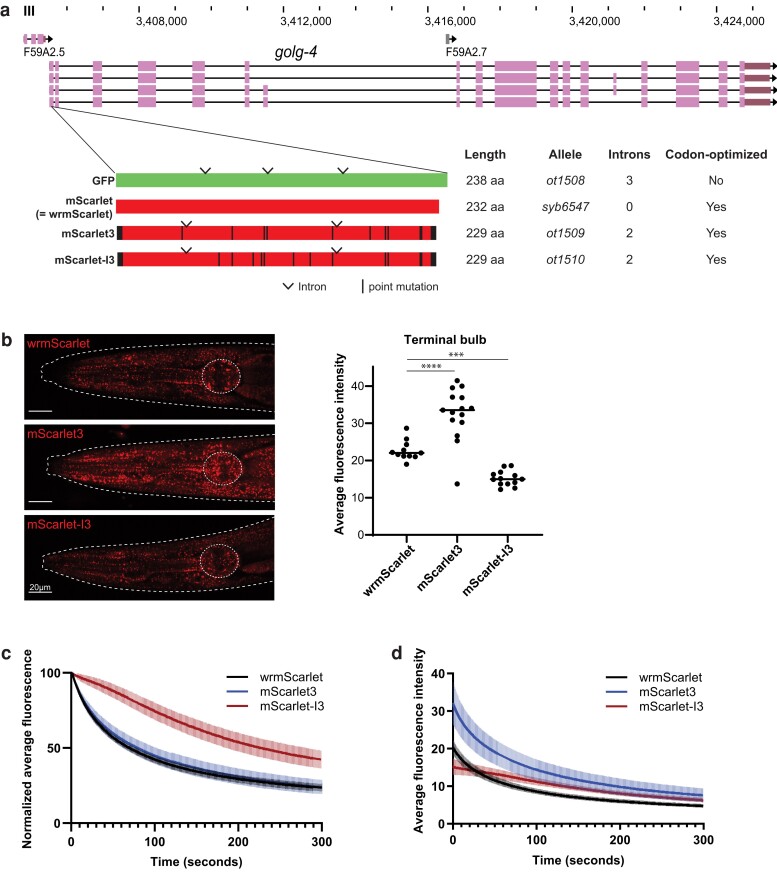
Analogous CRISPR-mediated insertion of fluorophores at the N-terminus of the GOLG-4 locus. a) WormBase genome browser snapshot of the *golg-4* locus and the site of CRIPSR fluorophore insert. The operon on which *golg-4* is located is schematically indicated and includes a third gene located further downstream that is not shown here. Codon-optimized fluorophores are schematized below: derivatives of mScarlet—mScarlet3 and mScarlet-I3—show amino acid mutations relative to mScarlet in black. GFP cloned from pPD95.75 expression plasmid is also included for comparison. Introns are also annotated and were added as recommended by the MPI *C. elegans* codon adapter (Redemann *et al.* 2011). b) Representative expression of red fluorophore-tagged GOLG-4 in the head is shown. Average fluorescence intensity of the pharyngeal terminal bulb (outlined in dotted white circle) of individual animals is quantified and plotted on the right, and the median values are indicated. ***P < 0.001, ****P < 0.0001, two-tailed t test. c and d) Photobleaching of the red fluorophores were measured through a time course of repeated imaging of RFP-tagged GOLG-4 expression in the same region and using the same imaging parameters as in panel b. Worms were imaged once per second over 300 s, and the fluorescence intensity was plotted over time, relative to starting values (c), or as average fluorescence intensity of the region of interest (d). Means and standard deviations of at least 10 individuals per strain are shown.

In the course of analyzing *C. elegans* homologs of Golgi apparatus-associated golgin proteins (Munro 2011), we inserted the commonly used, codon-optimized, but intron-free wrmScarlet fluorophore (El Mouridi *et al.* 2017) at the N-terminus of GOLG-4, the *C. elegans* ortholog of the trans Golgi-associated coiled-coil golgin protein golgin-245 () (Fig. 1a). We noted that fluorophore signals were clearly detectable postembryonically, but barely visible during embryogenesis. This observation provided us with the impetus to compare the properties of wrmScarlet to the new RFP variants mScarlet3 and mScarlet-I3. To this end, we designed *C. elegans* codon-optimized, artificial intron-containing mScarlet3 and mScarlet-I3 coding sequences. Using CRISPR/Cas9 genome editing, these fluorophores were inserted at the same N-terminal *golg-4* location as wrmScarlet (Fig. 1a).

In all 3 reporter strains, tagged GOLG-4 is localized to puncta throughout the animal and is particularly bright in the gland cells of the posterior pharyngeal bulb (Fig. 1b). These localization patterns recapitulate the GFP-tagged GOLG-4, and these worms are viable with no notable effects on growth or morphology, suggesting that these RFP tags do not interfere with protein localization or function in these strains. To directly compare the brightness of these RFPs expressed in vivo, we quantified average fluorescence intensity of the posterior bulb in these CRISPR-tagged young adult worms. We find that mScarlet3 is about 50% brighter than wrmScarlet, while mScarlet-I3 is about 30% dimmer (Fig. 1b). Based on the documented effect of 5′ introns on protein expression levels (Crane *et al.* 2019), adding a more proximal 5′ intron into the canonical, intronless wrmScarlet may increase wrmScarlet::GOLG-4 expression. However, since *golg-4* is the second gene in an operon (Fig. 1a), in which the first gene contributes 5′ introns, we do not consider this to be a major impediment to our comparisons of fluorescence intensities. In any case, these results correlate with in vitro spectroscopic characteristics of these fluorophores but differ from brightness characterized in mammalian cell culture(Gadella *et al.* 2023).

We also assayed the photostability of these RFPs by measuring the brightness of the GOLG-4-tagged strains during a time course with repeated confocal imaging once per second for 5 min. We find that wrmScarlet and mScarlet3 have very similar photostability, decreasing to 50% of initial brightness by 70 and 80 s, respectively (Fig. 1c). In contrast, mScarlet-I3 photobleached much slower, decreasing to 50% of initial brightness after 226 s of imaging (Fig. 1c). Given the different starting brightness of the RFPs, we also plotted the fluorescence intensity over time. We find that mScarlet3 remains the brightest of the 3 RFPs throughout the time course despite photobleaching faster than mScarlet-I3 (Fig. 1d). In addition, mScarlet-I3 brightness surpasses that of wrmScarlet after 28 time points due to decreased extent of photobleaching (Fig. 1d). These results suggest that mScarlet3 and mScarlet-I3 may be superior compared to wrmScarlet for live imaging or extended laser exposure.

### mScarlet-I3 signal accumulates at comparable rates to GFP

As a benchmark for relative maturation speed of the RFPs, we compared their fluorescent expression dynamics against GFP by visualizing GOLG-4 accumulation in the developing *C. elegans* embryo. Embryos expressing GOLG-4 with various RFP tags were imaged at specific stages over the course of embryo development from the 4-cell stage to the 4-fold stage, a period of about 9 h, and compared to GOLG-4 similarly tagged with GFP, derived from the *C. elegans* expression plasmid from the Fire vector kit, pPD95.75 (Fig. 1a). This GFP tag, though not codon-optimized for *C. elegans*, remains very commonly used and serves as a benchmark for our comparisons.

**Fig. 2.**
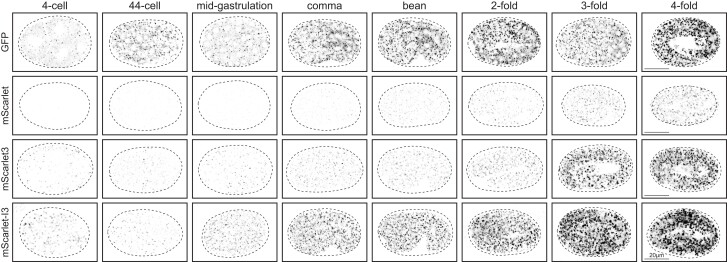
Expression of fluorophore-tagged GOLG-4 through embryonic development. Representative images of single focal planew through the middle of each embryo are shown, comparing the expression dynamics between the mScarlet variants and GFP. Strains are those shown in Fig. 1. For comparison to GFP, imaging setting was chosen to match the fluorescence intensity of GFP::GOLG-4 to that of mScarlet-I3::GOLG-4 in the adult, quantified in Fig. 1. Eight embryo at developmental stages from the 4-cell stage to the 4-fold stage, spanning most of embryogenesis, are represented in columns and compared across fluorophores, in rows. Embryo stages were determined by DIC (not shown), and eggshells are traced in dashed lines. The detected expression of GFP::GOLG-4 and mScarlet-I3::GOLG-4 in 4 cell stage embryos suggests maternal contribution of either protein or transcript.

We found that GFP::GOLG-4 puncta were detectable at low levels in the 4-cell embryo, and accumulates steadily at each subsequent stage captured throughout embryogenesis to the 4-fold stage (Fig. 2). In contrast, wrmScarlet::GOLG-4 was not visible for several hours in the early embryo. wrmScarlet signal accumulates extremely slowly, is detected at low levels after onset of expression, and remains weakly expressed through the end of the time course. These observations are consistent with test data from mammalian cells, where mScarlet has a maturation time of nearly 3 h at 37°C (Gadella *et al.* 2023).

Despite being a brighter fluorophore than wrmScarlet with a much faster maturation time in cell lines, mScarlet3::GOLG-4 fluorescence was also extremely slow to accumulate, detected only at low levels until the 3-fold stage (Fig. 2). Evidently, the vast improvement of mScarlet3 maturation time—37 min in mammalian cells (Gadella *et al.* 2023)—had limited effects on expression dynamics in *C. elegans*.

In stark contrast, the onset of mScarlet-I3::GOLG-4 expression is comparable to GFP::GOLG-4. Like the GFP reporter allele, it is also visible at the 4-cell stage, and also accumulates readily and steadily over the captured time points through embryo development (Fig. 2). Moreover, mScarlet-I3 signal intensity surpasses that of wrmScarlet and mScarlet3 by the 3-fold stage, despite being a dimmer fluorophore. Indeed, mScarlet-I3::GOLG-4 signal accumulates to higher levels much faster than its RFP counterparts.

### mScarlet-I3 behavior in other cells and subcellular locales

**Fig. 3.**
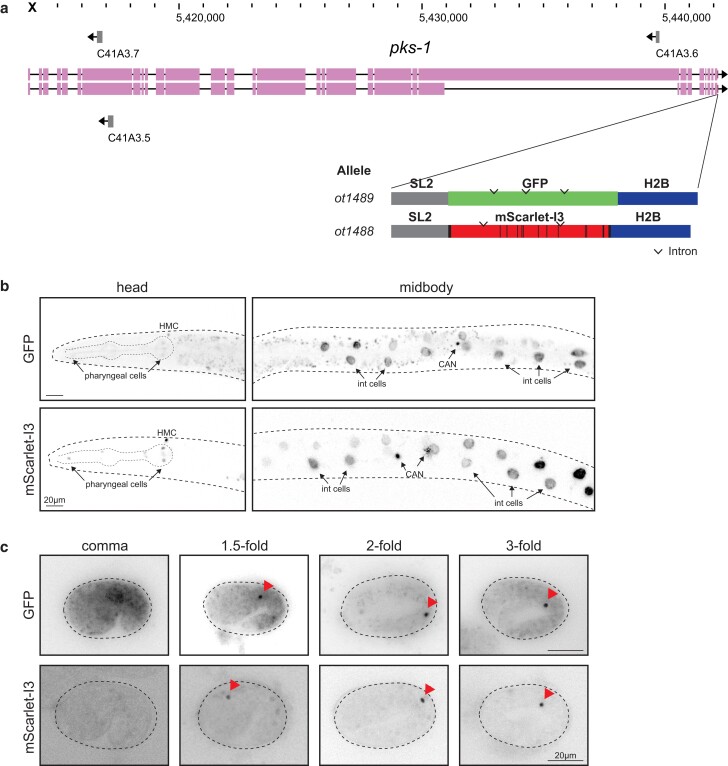
Expression of *pks-1::SL2::GFP::H2B* and *pks-1::SL2::mScarlet-I3::H2B*. a) WormBase genome browser snapshot of the *psk-1* locus and the site of CRISPR-mediated insertion at the C-terminus, after the stop codon. Schematic of fluorophore constructs is shown below. b) Expression of fluorophores in the L3 larval head and midbody. Both GFP and mScarlet-I3 reporters are expressed in the nuclei of CANL/R and intestinal (int) cells. Lower expression is also observed in several nuclei within the pharynx. An additional nucleus in the head, presumed to be the head mesodermal cell (HMC), is also labeled. Images are of a single plane containing 1 of the 2 CAN nuclei in focus. c) Expression of fluorophores in various stages of the embryo. Focal plane containing 1 of the 2 nuclei is shown for each embryo. Fluorescent signal can be visualized from the comma stage onwards, for both GFP and mScarlet-I3 constructs (arrowheads). Note the *pks-1* reporter constructs specifically label CAN nuclei at these embryonic stages shown.

To independently validate these findings in different cellular and subcellular contexts, we tagged another locus, *pks-1*, encoding a polyketide synthase (Feng *et al.* 2021), with GFP or mScaret-I3. In this case, we did not fuse the fluorophores directly to the gene, but used a standard polycistronic cassette approach in which the fluorophores were separated from the tagged gene with a trans-splicing SL2 signal and targeted to the nucleus by addition of a histone 2B (*his-44*) cassette (Fig. 3a). Late larval stage animals carrying *pks-1::SL2::mScarlet-I3::H2B* or *pks-1::SL2::GFP::H2B* show fluorophore expression in the nuclei of the CAN neuron pair (consistent with previous findings; (Feng *et al.* 2021)) and in cells of the alimentary system, including intestinal cells as well as pharyngeal cells (Fig. 3b and c). Notably, pharyngeal cells showed brighter signal with the mScarlet-I3 reporter compared to the GFP reporter; the mScarlet-I3 signal also appeared crisper because of lesser background fluorescence (Fig. 3b). In the intestine, the signal-to-noise ratio is even more improved with the mScarlet-I3 reporter since the characteristic autofluorescent puncta of the intestine in the green channel are nearly absent when imaging in the red channel. As a result, the adjacent CAN nuclei in the midbody of the worm are much more easily discernable when marked with mScarlet-I3 (Fig. 3b).

Next, these animals were analyzed for onset of fluorophore expression in the embryo and found to display comparable expression dynamics: fluorescently-labeled CAN nuclei are not visible at the bean stage with either the GFP or mScarlet-I3 fluorophore, but become clearly detectable shortly after by the comma stage and through the rest of embryo development (Fig. 3c). We again observe much less background autofluorescence when imaging the mScarlet-I3 reporter compared to the GFP reporter, especially at earlier embryonic stages (Fig. 3c).

Taken together, mScarlet-I3 appears superior to GFP in terms of increased signal intensity, decreased background particularly in the intestine, while exhibiting a comparable maturation time to GFP.

### mScarlet-I3::UNC-75 as a red pan-neuronal marker

**Fig. 4.**
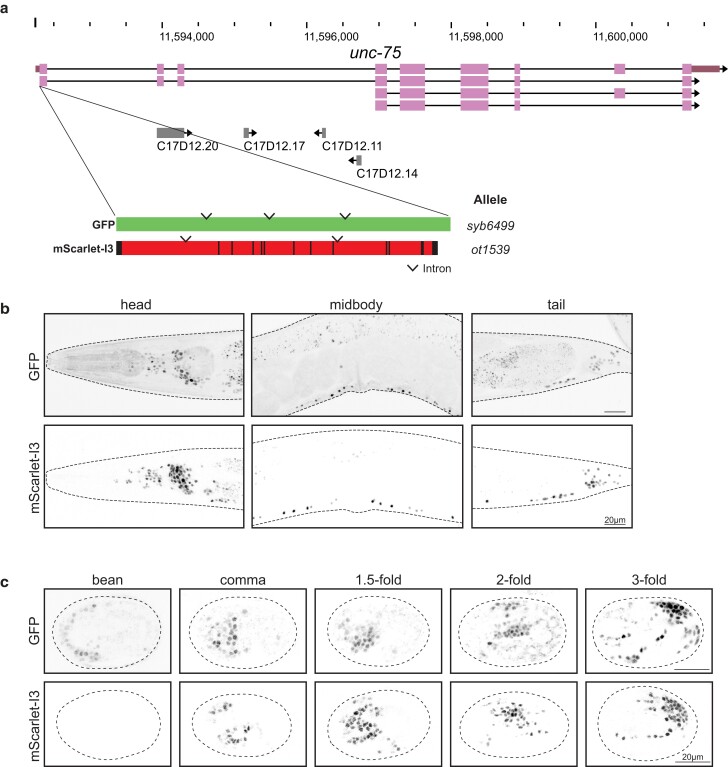
Expression of fluorophore-tagged UNC-75, comparing GFP to mScarlet-I3. a) WormBase genome browser snapshot of the *unc-75* locus, showing the site of CRISPR-mediated insertion at the N-terminus. Fluorophore constructs are the same as in previous figures. The fluorophores were inserted at the N-terminus to avoid disruption of a C-terminal nuclear localization sequence (Loria *et al.* 2003). b) Expression of fluorophore-tagged UNC-75 in the adult worm. Images are maximum intensity projections through the head, midbody, and tail sections, capturing dense regions of neuron nuclei. c) Expression of fluorophore-tagged UNC-75 at 5 stages in embryo development, determined by DIC. Single *Z* planes through the approximate midpoint of each embryo are shown, and eggshells are traced with dashed lines.

As an additional case study, we translationally tagged *unc-75*, a CELF family RNA-binding protein expressed specifically in all neuronal nuclei but no other cell types (Loria *et al.* 2003), with both GFP (Leyva-Diaz *et al*. 2024) and mScarlet-I3 (Fig. 4a) and characterized onset of expression of the resulting reporter alleles. We observe pan-neuronal nuclear expression of both fluorophores throughout the adult worm. These engineered worms appear morphologically wild type with no apparent defects in locomotion, suggesting that the tagged protein remains functional. Consistent with the above observations, we also observe improved signal-to-noise ratio of the mScarlet-I3 reporter compared to the GFP reporter, particularly resulting from decreased autofluorescence in the intestine associated with a longer wavelength excitation laser when imaging mScarlet-I3 (Fig. 4b).

We observe the earliest onset of GFP::UNC-75 expression at low levels around the bean stage (Fig. 4c). GFP signal accumulates quickly in subsequent embryonic stages. In comparison, mScarlet-I3::UNC-75 levels were just below detection threshold at the bean stage, but become visible shortly after, accumulating to comparable levels by the comma stage (Fig. 4c). Hence, in this case, expression of mScarlet-I3 slightly lags behind that of GFP. However, we also note that like in adults, mScarlet-I3::UNC-75 expression in the embryo has less nonspecific background fluorescence signal and better signal-to-noise ratio compared to GFP::UNC-75.

## Discussion

We described here that mScarlet-I3 is the fastest maturing mScarlet-based fluorescent marker currently tested in *C. elegans*, making it the prime choice for usage if maturation time is an important factor. mScarlet-I3 is not quite as bright as mScarlet3, but given its overall brightness combined with low background fluorescence in the red channel, this only becomes an issue if gene or protein expression are particularly low. Although brightness of these RFPs was only compared at the GOLG-4 locus in this study, and there may be gene-dependent variations, these data suggest that mScarlet3 is exceptionally bright, and may be more useful for visualizing especially lowly expressed genes when expression dynamics are not of concern.

Two additional factors should be considered relating to the use of these fluorophores in *C. elegans*, as mentioned in the results. First, the GFP used throughout these experiments is the commonly used construct from the Fire vector kit, pPD95.75, which was not explicitly codon-adapted for optimal *C. elegans* expression. We compare expression of our RFPs against this particular GFP tag as a benchmark due to its popularity in the community. However, a codon-optimized GFP may increase its expression levels further.

Second, the wrmScarlet used in the GOLG-4 comparisons lack introns, compared to its synthetic intron-containing mScarlet3 and mScarlet-I3 tag counterparts. Since it has previously been shown that the position of the first 5′ intron can increase protein expression (Crane *et al.* 2019), it could be argued that adding introns to our N-terminal wrmScarlet tag may boost overall expression level of the tagged protein. However, since the *golg-4* gene, with which we conducted our comparisons, is the second gene in an operon that is preceded by an intron-containing gene, we consider the intronless nature of mScarlet to be a minor issue in our experimental setup.

In conclusion, we have shown here in 3 cases that the new RFPs mScarlet3 and mScarlet-I3 both have clearly measurable advantages over wrmScarlet in terms of expression dynamics, and over GFP in terms of signal-to-noise ratio. These fluorophores will expand the utility of RFPs for fluorescent imaging in *C. elegans*.

## Supplementary Material

iyae126_Supplementary_Data

## Data Availability

All strains have been deposited at the CGC. Supplemental material available at GENETICS online.
