# Supplementary material for: Comparative analysis of new mScarlet-based red fluorescent tags in Caenorhabditis elegans
Source: Genetics. 2024 Aug 6;228(2):iyae126. doi: 10.1093/genetics/iyae126 (PMC11457934; doi:10.1093/genetics/iyae126)
Supplement: iyae126_Supplementary_Data [file iyae126_supplementary_data.docx]

**Supplemental file S1: Fluorophore sequences**

The *C. elegans* codon-optimized mScarlet-3 and mScarlet-I3 sequences below have been cloned into the pPD95.75 backbone in place of GFP and have been deposited to Addgene with the following plasmid names:

pWXC077: mScarlet3

pWXC078: mSarlet-I3

Overhang sequences used to replace GFP in pPD95.75 were:

5’-gacccttggagggtaccggtagaaaaa-3’

5’-cattcgtagaattccaactg-3’

**XXXX** coding sequence

xxxx introns

**>mScarlet3**

**ATGGACTCCACCGAGGCCGTCATCAAGGAGTTCATGCGTTTCAAGGTCCACATGGAGGGATCCATGAACGGACACGAGTTCGAGATCGAGGGAGAGGGAGAGGGACGTCCATACGAGGGAACCCAAACCGCCAAGCTCCGTGTCACCAAG**gtaagtttaaacatatatatactaactaaccctgattatttaaattttcag**GGAGGACCACTCCCATTCTCCTGGGACATCCTCTCCCCACAATTCATGTACGGATCCCGTGCCTTCACCAAGCACCCAGCCGACATCCCAGACTACTGGAAGCAATCCTTCCCAGAGGGATTCAAGTGGGAGCGTGTCATGAACTTCGAGGACGGAGGAGCCGTCTCCGTCGCCCAAGACACCTCCCTCGAGGACGGAACCCTCATCTACAAGGTCAAGCTCCGTGGAACCAACTTCCCACCAGACGGACCAGTCATGCAAAAGAAGACCATGGGATGGGAGGCCTCCACCGAGCGTCTCTACCCAGAGGACGTCGTCCTCAAG**gtaagtttaaacagttcggtactaactaaccatacatatttaaattttcag**GGAGACATCAAGATGGCCCTCCGTCTCAAGGACGGAGGACGTTACCTCGCCGACTTCAAGACCACCTACCGTGCCAAGAAGCCAGTCCAAATGCCAGGAGCCTTCAACATCGACCGTAAGCTCGACATCACCTCCCACAACGAGGACTACACCGTCGTCGAGCAATACGAGCGTTCCGTCGCCCGTCACTCCACCGGAGGATCCGGAGGATCCTAA**

**>mScarlet-I3**

**ATGGACTCCACCGAGGCCGTCATCAAGGAGTTCATGCGTTTCAAGGTCCACATGGAGGGATCCATGAACGGACACGAGTTCGAGATCGAGGGAGAGGGAGAGGGACGTCCATACGAGGGAACCCAAACCGCCAAGCTCAAGGTCACCAAG**gtaagtttaaacatatatatactaactaaccctgattatttaaattttcag**GGAGGACCACTCCCATTCTCCTGGGACATCCTCTCCCCACAATTCATGTACGGATCCCGTGCCTTCATCAAGCACCCAGCCGACATCCCAGACTACTGGAAGCAATCCTTCCCAGAGGGATTCAAGTGGGAGCGTGTCATGATCTTCGAGGACGGAGGAACCGTCTCCGTCACCCAAGACACCTCCCTCGAGGACGGAACCCTCATCTACAAGGTCAAGCTCCGTGGAGGAAACTTCCCACCAGACGGACCAGTCATGCAAAAGCGTACCATGGGATGGGAGGCCTCCACCGAGCGTCTCTACCCAGAGGACGTCGTCCTCAAG**gtaagtttaaacagttcggtactaactaaccatacatatttaaattttcag**GGAGACATCAAGATGGCCCTCCGTCTCAAGGACGGAGGACGTTACCTCGCCGACTTCAAGACCACCTACAAGGCCAAGAAGCCAGTCCAAATGCCAGGAGCCTTCAACATCGACCGTAAGCTCGACATCACCTCCCACAACGAGGACTACACCGTCGTCGAGCAATACGAGCGTTCCGTCGCCCGTCACTCCACCGGAGGATCCGGAGGATCCTAA**

**>wrmScarlet**

**GTCAGCAAGGGAGAGGCAGTTATCAAGGAGTTCATGCGTTTCAAGGTCCACATGGAGGGATCCATGAACGGACACGAGTTCGAGATCGAGGGAGAGGGAGAGGGACGTCCATACGAGGGAACCCAAACCGCCAAGCTCAAGGTCACCAAGGGAGGACCACTCCCATTCTCCTGGGACATCCTCTCCCCACAATTCATGTACGGATCCCGTGCCTTCACCAAGCACCCAGCCGACATCCCAGACTACTACAAGCAATCCTTCCCAGAGGGATTCAAGTGGGAGCGTGTCATGAACTTCGAGGACGGAGGAGCCGTCACCGTCACCCAAGACACCTCCCTCGAGGACGGAACCCTCATCTACAAGGTCAAGCTCCGTGGAACCAACTTCCCACCAGACGGACCAGTCATGCAAAAGAAGACCATGGGATGGGAGGCCTCCACCGAGCGTCTCTACCCAGAGGACGGAGTCCTCAAGGGAGACATCAAGATGGCCCTCCGTCTCAAGGACGGAGGACGTTACCTCGCCGACTTCAAGACCACCTACAAGGCCAAGAAGCCAGTCCAAATGCCAGGAGCCTACAACGTCGACCGTAAGCTCGACATCACCTCCCACAACGAGGACTACACCGTCGTCGAGCAATACGAGCGTTCCGAGGGACGTCACTCCACCGGAGGAATGGACGAGCTCTACAAG**

(no start or stop included because this sequence was inserted at the C-terminus, before the stop codon, of the golg-4 gene)

**>GFP** (from pPD95.75)

**Atgagtaaaggagaagaacttttcactggagttgtcccaattcttgttgaattagatggtgatgttaatgggcacaaattttctgtcagtggagagggtgaaggtgatgcaacatacggaaaacttacccttaaatttatttgcactactggaaaactacctgttccatgg**gtaagtttaaacatatatatactaactaaccctgattatttaaattttcag**ccaacacttgtcactactttctgttatggtgttcaatgcttctcgagatacccagatcatatgaaacggcatgactttttcaagagtgccatgcccgaaggttatgtacaggaaagaactatatttttcaaagatgacgggaactacaagacacgt**aagtttaaacagttcggtactaactaaccatacatatttaaattttcaggt**gctgaagtcaagtttgaaggtgatacccttgttaatagaatcgagttaaaaggtattgattttaaagaagatggaaacattcttggacacaaattggaatacaactataactcacacaatgtatacatcatggcagacaaacaaaagaatggaatcaaagtt**gtaagtttaaacatgattttactaactaactaatctgatttaaattttcag**aacttcaaaattagacacaacattgaagatggaagcgttcaactagcagaccattatcaacaaaatactccaattggcgatggccctgtccttttaccagacaaccattacctgtccacacaatctgccctttcgaaagatcccaacgaaaagagagaccacatggtccttcttgagtttgtaacagctgctgggattacacatggcatggatgaactatacaaatag**
